# Supplementary material for: Intravenous and intracranial GD2-CAR T cells for H3K27M+ diffuse midline gliomas
Source: Nature. 2024 Nov 13;637(8046):708–15. doi: 10.1038/s41586-024-08171-9 (PMC11735388; doi:10.1038/s41586-024-08171-9)
Supplement: Supplementary file 2 — Reporting Summary [file 41586_2024_8171_MOESM2_ESM.pdf]

nature portfolio

Corresponding author(s): Michelle Monje, Sneha Ramakrishna, Crystal Mackall

Last updated by author(s): Sep 5, 2024

Reporting Summary

Nature Portfolio wishes to improve the reproducibility of the work that we publish. This form provides structure for consistency and transparency in reporting. For further information on Nature Portfolio policies, see our Editorial Policies and the Editorial Policy Checklist.

Statistics

For all statistical analyses, confirm that the following items are present in the figure legend, table legend, main text, or Methods section.

n/a	Confirmed	
☐	☒	The exact sample size (*n*) for each experimental group/condition, given as a discrete number and unit of measurement
☐	☒	A statement on whether measurements were taken from distinct samples or whether the same sample was measured repeatedly
☐	☒	The statistical test(s) used AND whether they are one- or two-sided<br>*Only common tests should be described solely by name; describe more complex techniques in the Methods section.*
☐	☒	A description of all covariates tested
☐	☒	A description of any assumptions or corrections, such as tests of normality and adjustment for multiple comparisons
☐	☒	A full description of the statistical parameters including central tendency (e.g. means) or other basic estimates (e.g. regression coefficient) AND variation (e.g. standard deviation) or associated estimates of uncertainty (e.g. confidence intervals)
☐	☒	For null hypothesis testing, the test statistic (e.g. *F*, *t*, *r*) with confidence intervals, effect sizes, degrees of freedom and *P* value noted<br>*Give P values as exact values whenever suitable.*
☒	☐	For Bayesian analysis, information on the choice of priors and Markov chain Monte Carlo settings
☒	☐	For hierarchical and complex designs, identification of the appropriate level for tests and full reporting of outcomes
☒	☐	Estimates of effect sizes (e.g. Cohen's *d*, Pearson's *r*), indicating how they were calculated

Our web collection on statistics for biologists contains articles on many of the points above.

Software and code

Policy information about availability of computer code

Data collection	n/a
Data analysis	PRISM (version 10) software was used for statistical analyses

For manuscripts utilizing custom algorithms or software that are central to the research but not yet described in published literature, software must be made available to editors and reviewers. We strongly encourage code deposition in a community repository (e.g. GitHub). See the Nature Portfolio guidelines for submitting code & software for further information.

Data

Policy information about availability of data

All manuscripts must include a data availability statement. This statement should provide the following information, where applicable:

- Accession codes, unique identifiers, or web links for publicly available datasets
- A description of any restrictions on data availability
- For clinical datasets or third party data, please ensure that the statement adheres to our policy

All raw data are available in the source data file.

Research involving human participants, their data, or biological material

Policy information about studies with human participants or human data. See also policy information about sex, gender (identity/presentation), and sexual orientation and race, ethnicity and racism.

Reporting on sex and gender	Of the 13 patients enrolled, 7 patients were female and 6 patients were male
Reporting on race, ethnicity, or other socially relevant groupings	n/a
Population characteristics	Enrollment began in June 2020 and the data cutoff was December 1, 2023. Median age was 15 yrs (range 4-30 yrs), and seven patients were female. Ten patients had DIPG and three had sDMG. Two patients (Patients 002 and 011) were removed from study prior to treatment due to rapid tumor progression and a decline in performance status rendering them ineligible to protocol-directed therapy.
Recruitment	Patients on this Phase 1 clinical trial were recruited through physician and self-referral. PAtents were recruited from throughout the United States, and referrals came from both academic centers and community health centers.
Ethics oversight	The Stanford University IRB approved this clinical study.

Note that full information on the approval of the study protocol must also be provided in the manuscript.

Field-specific reporting

Please select the one below that is the best fit for your research. If you are not sure, read the appropriate sections before making your selection.

☒ Life sciences ☐ Behavioural & social sciences ☐ Ecological, evolutionary & environmental sciences

For a reference copy of the document with all sections, see nature.com/documents/nr-reporting-summary-flat.pdf

Life sciences study design

All studies must disclose on these points even when the disclosure is negative.

Sample size	Arm A followed a 3+3 dose escalation design, starting at IV Dose Level 1 (IV DL1). Disease cohorts were analyzed separately, but the safety in subjects with DIPG informed dose escalation for subjects with spinal DMG, but not vice-versa. The rationale for this was concerns about enrollment speed (pediatric spinal DMG is less common than pediatric DIPG) and lower risk for CAR-inflammation associated brain herniation (due to the location of their tumor). The study also allowed up to 6 subjects to be replaced as inevaluable because they were unable to meet the protocol-defined eligibility to receive GD2CART cells to account for significant disease progression post-enrollment and prior to treatment. In total, Arm A enrolled 11 subjects (with 11 successful manufacturing runs) and treated 7. Four subjects (3 subjects with DIPG, 1 subject with spinal DMG) were enrolled on IV DL1 and three subjects treated, with 0 of 3 subjects with DIPG experiencing DLT. Because safety in DIPG informed spinal DMG cohort, both groups dose escalated to IV DL2. In IV DL2, 1 of 3 subjects with DIPG experienced DLT, necessitating expansion to 6 subjects with DIPG. Concurrent enrollment of subjects with spinal DMG to IV DL2 resulted in 1 of 2 subjects with spinal DMG experiencing DLT. Ultimately, 2 of 6 subjects with DIPG and 1 of 2 subjects with spinal DMG experienced DLT, causing IV DL2 to exceed allowable DLT rate. All DLT cases were due to cytokine release syndrome (CRS). All inevaluable subjects in Arm A were ineligible to receive GD2CART cells on study because of disease progression.<br>Because CRS caused by systemic immune activation was cause of all DLT, we subsequently modified the protocol and began to test an ICV-only strategy route and schedule (Arms B and C) that would diminish the chances of dose-limiting CRS.
Data exclusions	No data were excluded for the 11 patients treated on trial. Two patients (Patients 002 and 011) were removed from study prior to treatment due to rapid tumor progression and a decline in performance status rendering them ineligible to protocol-directed therapy. One of these two patients was treated on an eIND and reported in Majzner et al., 2022 Nature.
Replication	ctDNA, and RT-PCR for CAR transgene were performed in triplicate, Cytokine analyses were performed in duplicate.
Randomization	No randomization was performed in this Phase 1 clinical trial, as this is a non-randomized early-phase trial.
Blinding	No blinding was performed in this Phase 1 clinical trial, as this is a non-blinded early-phase trial.

Reporting for specific materials, systems and methods

We require information from authors about some types of materials, experimental systems and methods used in many studies. Here, indicate whether each material, system or method listed is relevant to your study. If you are not sure if a list item applies to your research, read the appropriate section before selecting a response.

Materials & experimental systems

n/a	Involved in the study
☐	☒ Antibodies
☒	☐ Eukaryotic cell lines
☒	☐ Palaeontology and archaeology
☒	☐ Animals and other organisms
☐	☒ Clinical data
☒	☐ Dual use research of concern
☒	☐ Plants

Methods

n/a	Involved in the study
☒	☐ ChIP-seq
☐	☒ Flow cytometry
☐	☒ MRI-based neuroimaging

Antibodies

Antibodies used

Each row provides information in the following order: Antigen Fluorochrome Clone Supplier Part Number
CD3 FITC UCHT1 BioLegend 300406
CD8 PerCP Cy5.5 SK1 BD Pharmingen 565310
CD45 BV785 2D1 BioLegend 368528
CD4 BV711 RPA-T4 BioLegend 300558
CD95 BV650 DX2 BioLegend 305624
CD39 Bv605 A1 BioLegend 328236
Cell viability BV510 N/A Invitrogen L-34965
CD57 BV421 NK-1 BDBiosciences 563896
CCR7 BUV805 2L1A BDBiosciences 749673
CD45RA Alx700 HI100 BioLegend 304120
GD2CAR DyLight650 1A7 NCI Biological Resources Branch, Custom
CD14 PE-Cy7 63D3 BioLegend 367112
CD11b APC-Cy7 ICRF44 BioLegend 301352
CD33 PE-Dazzle WM53 BIolegend 303432
GD2 PE 14G2A BioLegend 357304

Validation

All antibodies were validated as reported by the manufacturer (references below) except 1A7. In the case of 1A7, the anti-GD2 CAR idiotype antibody, untransduced T cells were used as a biological control. To Determine the optimal concentration of antibody for staining in the CAR-FACS panel, each fluorochrome-conjugated antibody was titrated to determine the saturating amount of antibody needed to stain a test/ one million cells. Dose response curves for each antibody informed the saturating amount of fluorochrome-conjugated antibody for staining a million cells in 100 ml staining volume.
antigen and antibody validation reference:
CD3 Salmeron A et al.1991
CD8 Behjat et al. 2005
CD45 Knapp W et al.1989
CD4 Knapp W et al.1989
CD95 Schlossman S 1995
CD39 Aversa G. 1988
CD57 Abo T. et al.1981
CCR7 Birkenbach M. 1993
CD45RA Knapp W et al.1989
GD2-CAR Sen et al. 1997
CD14 Antonyshyn et al. 2022
CD11b Knapp W et al.1989
CD33 Knapp W et al.1989
GD2 Mujoo et al. 1989

Clinical data

Policy information about clinical studies
All manuscripts should comply with the ICMJE guidelines for publication of clinical research and a completed CONSORT checklist must be included with all submissions.

Clinical trial registration

NCT04196413

Study protocol

The trial protocol is provided in the supplementary information

Data collection

June 2020 to December 2023 at the Lucile Packard Children's Hospital at Stanford.

Outcomes

Primary objectives were to determine feasibility of manufacturing, assess safety and tolerability and identify a maximally tolerated and/or recommended phase 2 dose of IV GD2-CART following lymphodepleting chemotherapy in this population. Secondary objectives included preliminary assessment of benefit as measured by radiographic response on MRI and by clinical improvement. Another secondary objective was to assess the safety and benefit of subsequent ICV administrations of GD2-CAR T-cells

Plants

Seed stocks: n/a

Novel plant genotypes: n/a

Authentication: n/a

Flow Cytometry

Plots

Confirm that:

☒ The axis labels state the marker and fluorochrome used (e.g. CD4-FITC).

☒ The axis scales are clearly visible. Include numbers along axes only for bottom left plot of group (a 'group' is an analysis of identical markers).

☒ All plots are contour plots with outliers or pseudocolor plots.

☒ A numerical value for number of cells or percentage (with statistics) is provided.

Methodology

Sample preparation: PBMC were isolated from fresh whole blood by gradient centrifugation on ficoll (Ficoll paque Plus, GE Healthcare, SigmaAldrich). Two to five million PBMC were stained with fixable Live/Dead aqua (Invitrogen) amine-reactive viability stain. Cells were then preincubated with Fc block (trustain, Biolegend) for 5 min, then stained at room temperature with the following fluorochrome conjugated mAb in an 15-color, 17-parameter staining combination.

Instrument: LSR (BD Biosciences)

Software: Analysis was performed in FlowJo

Cell population abundance: At least 106 cells were acquired unless restricted by the number of cells isolated from 8 ml of whole blood or when acquiring CSF isolated cells. The assay limit of detection for cells calculated as 1 in 104 of total acquired PBMCs.

Gating strategy: CAR T-cells in CSF and PBMC: Singlets->viable cells->CD45+->CD14-, CD3+ -> CD4 or CD8
CAR positivity gated based on control PBMC.
Representative gating strategy is shown in Extended Data Figure 10.

☒ Tick this box to confirm that a figure exemplifying the gating strategy is provided in the Supplementary Information.

Magnetic resonance imaging

Experimental design

Design type: clinical studies

Design specifications: clinical studies

Behavioral performance measures: n/a

Acquisition

Imaging type(s): clinical studies

Field strength: 3T

Sequence & imaging parameters: T2 sequences used for volumetric quantification of tumor size and T2 sequences shown

Area of acquisition: Brain and Spine

Diffusion MRI ☐ Used ☒ Not used

Preprocessing

Preprocessing software	n/a
Normalization	n/a
Normalization template	n/a
Noise and artifact removal	n/a
Volume censoring	*Define your software and/or method and criteria for volume censoring, and state the extent of such censoring.*

Statistical modeling & inference

Model type and settings	n/a
Effect(s) tested	n/a

Specify type of analysis: ☒ Whole brain ☐ ROI-based ☐ Both

Statistic type for inference (See Eklund et al. 2016)	n/a
Correction	n/a

Models & analysis

n/a	Involved in the study
☒	☐ Functional and/or effective connectivity
☒	☐ Graph analysis
☒	☐ Multivariate modeling or predictive analysis
